# Machine learning for urban land use/ cover mapping: Comparison of artificial neural network, random forest and support vector machine, a case study of Dilla town

**DOI:** 10.1016/j.heliyon.2024.e39146

**Published:** 2024-10-12

**Authors:** Melion Kasahun, Abiyot Legesse

**Affiliations:** a Department of Geography and Environmental Studies, College of Social Science and Humanities, Dilla University, P.O. Box: 419, Dilla, Ethiopia; b Department of Geography and Environmental Studies, College of Social Science and Humanities, Borana University, P.O. Box: 85, Yabello, Ethiopia

**Keywords:** Accuracy assessment, Algorithm stability, ANN, Computing time, LULC, RF, SVM, Visual quality

## Abstract

*The ability to accurately classify land use/cover (LULC) is critical for environmental monitoring and land use planning. This study compares three machine learning algorithms: Artificial Neural Network (ANN),* Support *Vector Machine (SVM), and Random Forest (RF) for LULC classification using Google Earth images from the years 2006, 2014, and 2022. The objective of this study is to evaluate and identify the best classifier for LULC classification and change detection. Four LULC categories (Built-up, Open area, Farmland, and Agroforestry) were identified. The evaluation criteria included overall accuracy, kappa coefficient, producer's accuracy, user's accuracy, computing time, algorithm stability, and visual quality. The results showed that the RF algorithm outperformed both SVM and ANN algorithms with an average overall accuracy of 0.97, kappa coefficient of 0.98, producer's accuracy of 0.99, and user's accuracy of 0.97, surpassing the accuracies achieved by SVM (0.96, 0.97, 0.98, and 0.97) and ANN (0.89, 0.81, 0.94, and 0.88), with corresponding computing times of 6.33, 15, and 30 s. All classifiers performed stably with different training sizes. Visual quality assessment revealed that RF had the highest precision. Consequently, the built-up change detection result shows, the net change in built-up area between 2006 and 2022 was increased by 0.*74 Km^2^*for ANN, 1.*74 Km2 *for SVM, and 1.*66 Km^2^*for RF. The comparison reveals that the RF algorithm showcasing high precision in detecting change, consistent with the data (increased by 1.*65 Km^2^*) obtained from Dilla town land administration office. To validate the results, the study considered field surveys, reference images, local experts, and previous studies. Based on the findings, the study concludes that using RF classifier with an object-based approach is an effective way to map LULC and detect changes in the study area over time*. *Future researchers are recommended to utilize this effective algorithm for addressing LULC related problems in the study area.*

## 1 Introduction

Land use/cover (LULC) changes represent a significant global challenge that profoundly affects natural resource management. The expansion of urban areas encroaches upon farmlands, resulting in notable shifts in population distribution, industrial activities, and land use practices [1,2]. This rapid urbanization, coupled with associated LULC alterations, poses severe environmental implications, encompassing soil compaction and sealing, decline in organic matter content, loss of biodiversity, air and water pollution, global warming, as well as an increased frequency of natural disasters like floods, droughts, and wildfires [3,4]. Ethiopia, characterized by diverse landscapes and climates, is grappling with rapid urban population growth, which is contributing to the depletion of natural resources [5].

The town of Dilla in Ethiopia serves as a poignant example of how swift LULC changes jeopardize land and water resources, a situation exacerbated by climate change that alters water availability and distribution [6]. Achieving sustainable resource management necessitates a profound comprehension of LULC dynamics. While traditional methods for LULC mapping, such as field surveys, are known to be laborious and costly, scientists and researchers have increasingly turned to remote sensing for efficient data collection on LULC alterations across expansive territories [6,7].

Machine learning (ML) algorithms present a potent alternative, enabling automated and efficient classification of LULC from remotely sensed data. Cutting-edge computer algorithms like Artificial Neural Networks (ANN), Support Vector Machines (SVM), and Random Forest (RF), are leveraged to scrutinize satellite imagery and monitor LULC changes. Often, these algorithms are integrated with the object-based image classification approach, which dissects groups of pixels known as image objects [8]. Such an approach facilitates precise land cover classification and the determination of physical properties critical for investigating LULC transformations [8]. The evaluation of various algorithms' performance assures the provision of reliable and accurate information crucial for effective LULC management [9].

By comparing different classification algorithms and pinpointing the most efficient performer, the process of obtaining dependable results in terms of output quality and accuracy is streamlined. This study undertakes a comparative analysis of the outcomes and accuracies of the ANN, RF, and SVM algorithms to evaluate their efficacy in LULC classifications derived from satellite remote sensing images. Additionally, the study introduces a novel object-based machine learning approach that evidently enhances the accuracy of LULC classification.

The core objective of this research is to furnish planning authorities with valuable insights for selecting the most suitable classification algorithm for LULC classification. While the study concentrates on comparing the performance of three specific machine learning algorithms, it refrains from delving into the intricacies of urban planning strategies themselves. The primary aim is to identify the optimal machine learning approach for achieving precise LULC classification, which serves as a foundational tool for informed decision-making in urban planning.

Through offering a comprehensive comparison, this study equips planning authorities with the knowledge necessary to choose the most effective tool for LULC mapping. This, in turn, will contribute to the formulation of sustainable development strategies that strike a balance between urban expansion and environmental conservation.

## 2 Methods and materials

### 2.1 Description of the study area

**Fig. 1:**
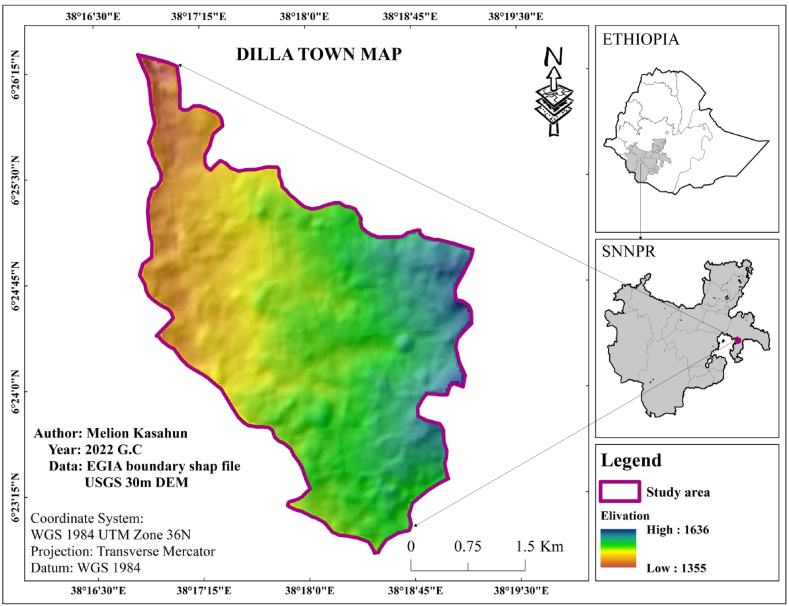
Location map of Dilla town (the study area).

Dilla town serves as the primary urban center within the Gedeo Zone of southern Ethiopia. Positioned strategically along the prominent roadway linking Addis Ababa, the capital of Ethiopia, to Nairobi, the capital of Kenya, the town plays a pivotal role in regional connectivity and transportation infrastructure. Geospatial data depicted in Fig. 1 precisely locates Dilla town within the coordinates of 6°23′15″- 6°26′15″ north latitude and 38°16′30″- 38°19′30″ east longitude. The town occupies an elevation of 1570 m above sea level, as illustrated in Fig. 1.

### 2.2 Methodology

**Fig. 2:**
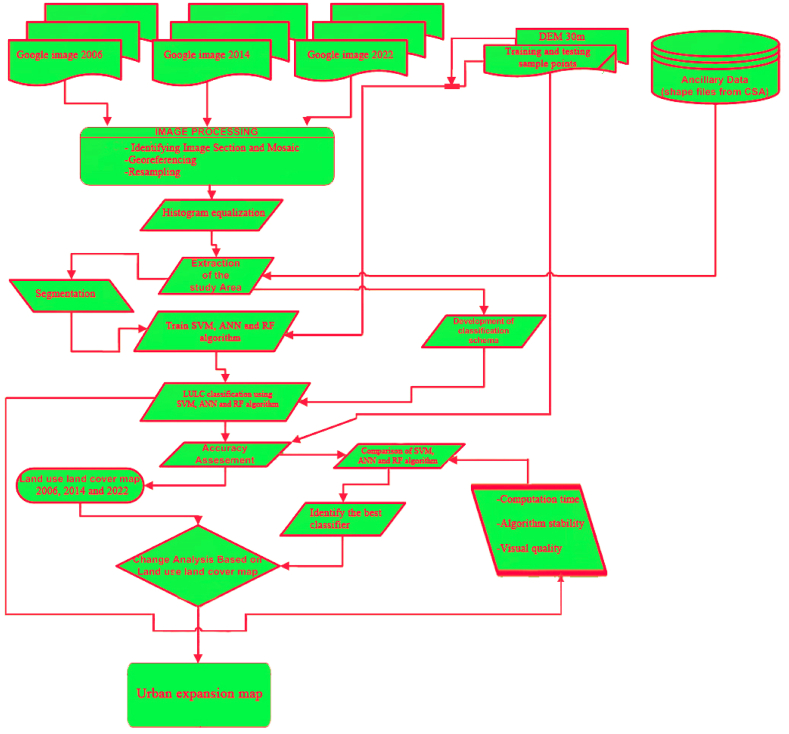
Methodological flow chart showing the data sources and methods of research analysis.

This section describes the analysis of satellite imagery to identify LULC changes in Dilla town over a 16-year period. Fig. 2 illustrates the overall methodology employed in this study.

### 2.3 Data sources and Acquisition

This research investigates the spatiotemporal variations in LULC utilizing advanced methodologies such as GIS and Remote Sensing. These techniques facilitated the analysis of spatial and temporal data pertaining to land attributes, such as built up characteristics, thereby unveiling temporal changes.

To collect these data, a combination of primary and secondary sources was employed. Primary sources encompassed satellite imagery, data acquired directly from field surveys, and insights provided by local experts. Furthermore, the study integrated pertinent information from prior research studies (secondary data).

#### 2.3.1 Satellite Imageries

The research extensively relied on Google Earth, an openly accessible platform providing diverse satellite images, as its primary data source. Google Earth is a virtual globe software by Google that enables users to explore high-resolution satellite imagery and aerial photographs of the Earth in both spatial and temporal dimensions. It presents multiple viewing options, including 3D visualization, and permits the integration of user-generated data such as shape files, placemarks, and lines.

These satellite images capture information at various spatial resolutions, offering detailed depictions of land features, and are captured periodically to ensure a high temporal resolution. These attributes render them invaluable tools for applications like LULC mapping, change detection over time, and project assessments. However, when images are downloaded from Google Earth, they may undergo resolution loss due to compression or the saving format utilized. To address this challenge, the study adopted a strategy of saving the images in a higher quality format by selecting a high-quality setting during the saving process. This method resulted in minimal deviation from the original 1m resolution. Subsequently, the image underwent resampling to precisely 1m to retain the slight variation observed and maintain a consistent resolution across all images, essential for overly analysis.

The study specifically employed three high-resolution Google satellite images captured 16 years apart (2006, 2014, and 2022). These years were chosen based on significant events that influenced land utilization in Dilla town.

### 2.4 Image preprocessing

Preprocessing of satellite images is a critical step prior to utilizing them for training and executing models for land cover analysis [[3], [4], [5], [6], [7], [8], [9], [10], [11]]. This process is essential for enhancing image quality by rectifying distortions and emphasizing significant features to facilitate a more straightforward analysis. The study utilized satellite images from Google Earth spanning the years 2006, 2014, and 2022, covering a 16-year duration.

#### 2.4.1 Geometric correction, image combination and extraction

This research employed georeferencing and geometric correction techniques to ensure accurate representation of surface features in the images. Subsequently, multiple images were mosaicked to generate a comprehensive picture of the study area. Regions of interest were then extracted from the mosaic for focused analysis using ArcMap 10.8 environment.

#### 2.4.2 Resampling for consistency

In image analysis, maintaining consistent resolution across all images is crucial for accurate comparisons. To achieve this uniformity in a study utilizing Google Earth images with varying resolutions, a resampling technique known as Nearest Neighbor was implemented in the ArcMap 10.8 environment. This technique assigns a pixel value based on its nearest neighbor in the image. It is a rapid process that conserves the original data values, rendering it suitable for discrete data such as LULC types. In this study, all images were resampled to a standardized pixel size of precisely 1 m.

#### 2.4.3 Enhancing image features (performed after preprocessing)

Image enhancement is a method used to enhance the visibility of specific features or land cover classes within an image [12]. This is achieved by adjusting the values of individual pixels [13]. The selection of an enhancement technique depends on the features to be highlighted.

One commonly used method is contrast stretching, which enhances the overall contrast between different elements in the image [14,15]. This study utilized two contrast stretching techniques: rescaling and histogram equalization. Both techniques were implemented in the ERDAS IMAGINE 2014 environment, enabling interactive adjustments during the enhancement process.

### 2.5 Development of classification scheme

The study began with existing information about the area. Then, examined a picture of the study area overall shape to gain a better understanding of its layout. Additionally, the study incorporated information from previous studies conducted in the area. This comprehensive approach facilitated the development of a land classification system with four categories: built-up areas, open areas, farmland, and agroforestry.

### 2.6 Image classification

The study used a technique called object-based classification to undertake classification of the images. First, the images were segmented into smaller areas. Then, a specific class (like forest, water, or building) was assigned to each segment based on its visual characteristics, such as color, shape, and texture. This approach is similar to how we visually interpret an image and identify objects within it.

#### 2.6.1 Object-oriented image analysis

While pixel-based methods are valuable for image classification, LULC categories are better represented by whole objects than individual pixels [16]. For this reason, this study utilizes object-based classification.

Object-based classification relies on identifying “image objects,” which are groups of similar pixels. To obtain these objects, a process called segmentation is typically used. Segmentation involves dividing a digital image into smaller, more meaningful segments [17]. The overall goal is to partition the image into these segments and then group them according to different algorithms.

There are various segmentation methods available, including thresholding, clustering, region-growing, split-and-merge, watershed transformation, model-based segmentation, trainable segmentation, and segment mean shift [[18], [19], [20], [21]]. This specific study employed segment mean shift for image clustering.

#### 2.6.2 Segmentation

In the field of image analysis, a technique called image segmentation is essential. It separates an image into distinct regions, such that each region groups pixels with similar characteristics [22]. This is a foundational step for analyzing objects within the image. This study used a specific segmentation technique called Segment Mean Shift (SMS) which is available in ArcGIS software [21,23]. SMS is an algorithm that builds regions by progressively incorporating neighboring pixels that share similar properties.

The SMS process relies on four user-defined settings: Spectral Detail, Spatial Detail, Band Indexes, and Minimum Segment Size. These settings influence the final segmentation outcome, determining the emphasis placed on spectral variations (color information) and the spatial proximity required for pixels to be grouped together [22]. When analyzing small and clustered features with similar spectral properties, higher values for Spectral Detail and Spatial Detail are preferred [24].

To create optimal segments for further analysis, various combinations of these parameters were tested, followed by visual evaluation of the segmentation quality. The results indicate that for small study areas with high-resolution images, using the maximum values for all four parameters yields the best outcome. This approach allows for more accurate identification of features and improves the classification process. Therefore, based on this visual verification, the maximum parameter settings were applied to all images for optimal segmentation.

#### 2.6.3 Developing training sites

**Fig. 3:**
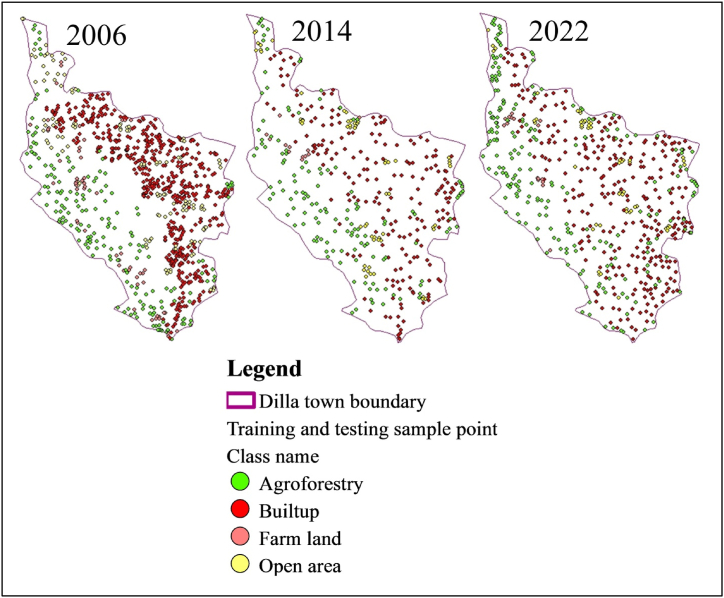
Training and testing sample points for each LULC class in the year 2006, 2014, and 2022.

Machine learning for land-use classification relies on well-defined training points. This study addressed this by using ground truth data collected via GPS for 2022 imagery. For images from 2006 to 2014, training points were chosen by integrating local community knowledge with Google Earth. The number of training points collected varied depending on the size of the LULC class. For larger LULC classes, a greater number of training points were collected, while smaller LULC classes had fewer training points collected. A total of 803, 405, and 578 samples were used for the years 2006, 2014, and 2022, respectively. Fig. 3 shows the training and testing sample points for each LULC class in the year 2006, 2014, and 2022, which were utilized in this study.

#### 2.6.4 Train classifier

##### 2.6.4.1 Support Vector machine

SVM are a type of machine learning algorithm used to classify data. It learns from labeled data (data with pre-assigned categories) and predicts the categories of new data points. Their main goal is to find the best decision boundary (a flat, dividing surface) in a high-dimensional space that separates the data points into their categories with the largest possible margin. This margin refers to the distance between the decision boundary and the closest data points on either side.

SVMs leverage kernel functions to create a suitable space for data analysis. While linear kernels are faster to compute, non-linear kernels often outperform them by enabling the algorithm to capture more complex relationships within the data [25]. To illustrate the core principles of SVM, let us consider the simpler case of a linear classifier, where data points are perfectly separable by a hyperplane in a high-dimensional space. Imagine a dataset where each data point (represented by xi) on a d-dimensional feature space corresponds to an object with a binary class label (either positive, denoted by 1, or negative, denoted by −1). The algorithm identifies the optimal hyperplane that maximizes the margin between the positive and negative data points.

While several data transformation techniques are available for SVMs, the radial basis function (RBF) kernel is one of the most commonly used [26]. In this study, the RBF is among the kernel functions utilized.

##### 2.6.4.2 Random forest

RF is a powerful approach to classification problems. They work by combining the predictions of many individual decision trees, like a group vote [27]. Each tree is built uniquely by using a random selection of features and a subset of training data. This randomness helps prevent the trees from becoming too similar and overfitting the data.

When a new data point comes along, each tree in the forest makes its own prediction about the class it belongs to. The final class label for the data point is decided by a majority vote: whichever class gets the most votes from the trees wins.

##### 2.6.4.3 Artificial neural network

ANN draws inspiration from the structure and function of the human brain [28]. The brain boasts billions of interconnected neurons that communicate through electrical signals. Similarly, ANN is comprised of artificial neurons organized in layers, interconnected with each other [29].

These artificial neurons function as information processors. They receive inputs from other neurons and generate outputs based on a specific mathematical function. A popular function employed in ANN is the sigmoid function, which introduces a non-linear element to the network. This function is crucial because it allows the network to learn intricate patterns within the data [30,31].

The core principle behind training ANN involves adjusting the strengths of the connections between neurons based on the data they are fed. This process empowers the network to learn and accurately classify new data points.

### 2.7 Accuracy assessment

In image analysis, accuracy assessment evaluates how well a classification performs by comparing the final classified image to a highly reliable reference map. As recommended by Ref. [3], an important step is to pick enough samples that represent each category on the map, and to make sure these samples are spread out evenly. A common rule is to use 50 samples for each class [32]. In this study, 50 sample points were used for each LULC class. The sample points were collected via GPS for the accuracy assessment of the 2022 generated map. For the generated maps of 2006 and 2014, accuracy assessment sample points were collected from Google Earth by integrating local community knowledge.

In assessing classification accuracy, two main types of scores are commonly used. The first type evaluates the overall performance of the classification, which includes metrics such as overall accuracy and the kappa coefficient. The second type, on the other hand, concentrates on the effectiveness of the classification within individual categories, such as LULC classes. This second type includes metrics such as producer accuracy (PA) and user accuracy (UA).

In this study, both overall performance assessment and single-category performance assessment were conducted to evaluate the performance of the machine learning algorithm.

### 2.8 Comparison of the algorithms performance and selection of the best method

The choice of classifier significantly impacts the outcome of a classification task. No single algorithm is a perfect fit for every situation due to variations in environmental factors and datasets [33]. To pick the most suitable method, researchers recommend considering several criteria, such as classification accuracy, computational requirements, running time, and robustness to imperfections in the training data [[33], [34], [35]].

This study assessed the algorithms based on the following factors: (a) accuracy of the classifications (overall accuracy, kappa coefficient, producer's accuracy and user's accuracy), (b) how long they take to run, (c) their stability, and (d) the visual quality of the outputs. It is important to note that accuracy is the most widely used metric for evaluating classification performance.

Ultimately, the algorithm that performed best was chosen for further analysis. This ensures efficient processing, minimizes errors, and leads to optimal results. By following this approach, the study can provide a solid foundation for making well-informed decisions.

## 3 Result and discussion

### 3.1 Segment mean shift

SMS is a type of clustering method that has been successful in computer vision and image processing [21]. It works by finding the peaks (called modes) of a density function in the image data. This is done iteratively by shifting the average (mean) of the data points within a defined area, which is why it is called mean shift.

**Fig. 4:**
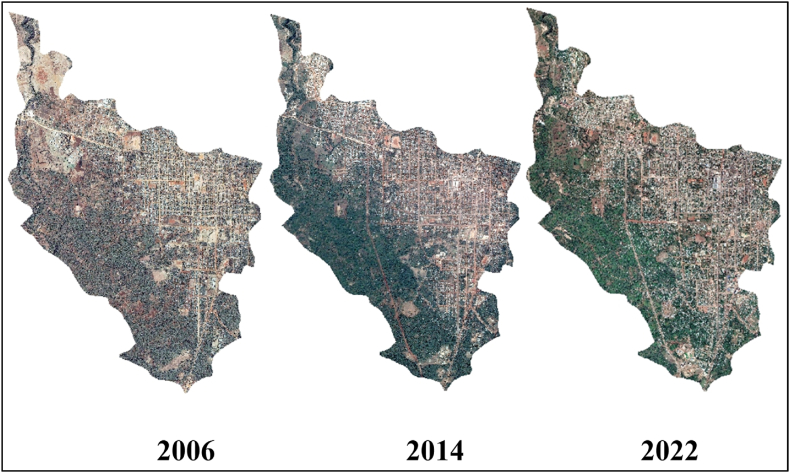
SMS-based segmentation result of 2006, 2014 and 2022 images.

When using SMS for image segmentation, several factors need to be considered for optimal results. These factors include the spectral details (colors), spatial details (arrangement of pixels), which image bands are used, and the minimum allowed size for each segment in pixels. The study referenced here employed SMS segmentation, and the results (shown in Fig. 4), suggest that using the most informative settings is particularly effective for small areas. Fig. 4 depicts the outcomes of SMS-based image segmentation for the year 2006, 2014, and 2022.

### 3.2 Implementation of machine learning classification for study area

This study focuses on identifying the most effective algorithm for classifying and detecting changes in urban LULC. To achieve this, the study defines four main LULC categories: Built-up, Open area, Farmland, and Agroforestry. These categories were chosen based on the specific research objectives. Three different algorithms - ANN, SVM, and RF - were then employed to classify the LULC data. By comparing the performance of these algorithms, the study aims to determine which one is best suited for urban LULC change detection.

#### 3.2.1 ANN classification

**Fig. 5:**
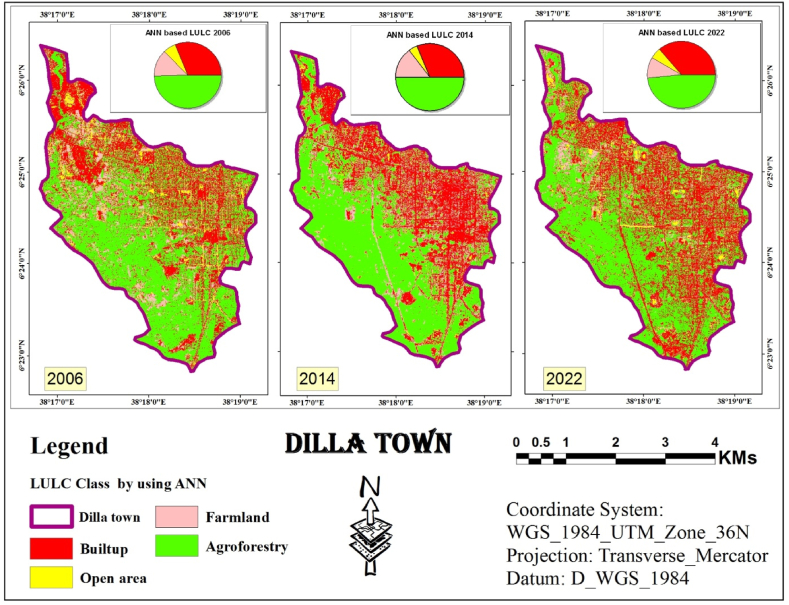
ANN classified image (2006, 2014 and 2022).

The study explored using ANN, a type of machine learning algorithm, for classifying digital images in remote sensing. The approach combines two techniques: object-based detection and clustering similar image pixels. These pixels are grouped based on information categories derived from the various spectral bands captured by a satellite image [[35], [36], [37]]. The final classification results obtained through the ANN are depicted in Fig. 5.

Following the object-based LULC classification using an ANN process, the statistics were calculated for the resulting LULC map. These statistics included the total area and the relative area (proportion) of each LULC class. This quantitative analysis helps to understand the composition of the entire area and the percentage each class occupies. Table 1 summarizes the processed results.

**Table 1:** Statistics of LULC classes Using ANN classifier.

LULC Class	2006		2014		2022	
	Area		Area		Area	
	(Km^2^)	%	(Km^2^)	%	(Km^2^)	%
**Built-up**	4.45	31.16	4.47	31.3	5.19	**36.34**
**Open area**	0.9	6.3	0.57	3.99	0.72	**5.04**
**Farm land**	1.82	12.75	2.1	14.71	1.45	**10.15**
**Agroforestry**	7.11	49.79	7.14	50	6.92	**48.46**
**Total Area (Km**^**2**^**)**	**14.28**	**100**	**14.28**	**100**	**14.28**	**100**

The land cover analysis of Dilla town spanning from 2006 to 2022 indicates that agroforestry consistently emerges as the predominant class, despite fluctuations observed in other categories. In 2006, agroforestry covered approximately half (49.79 %) of the total area (7.11 km2), as depicted in Fig. 5. This category comprised various trees, shrubs, and agricultural land with sparse vegetation. The second most prevalent class was the built-up area, which accounted for 31.16 % (4.45 km2) and was dispersed across the study area. Farmland ranked third, occupying 12.75 % (1.82 km2) and was predominantly concentrated in the northwestern and western regions. The remaining area (0.19 km2), representing 6.30 %, was classified as open space, marked by scattered yellow patches in Fig. 5.

The land cover distribution in 2014 exhibited a similar trend, with agroforestry maintaining its dominance (7.14 km2, 50 % share) as depicted in Fig. 5. There was a slight uptick in the built-up area (4.47 km2, 31.30 %), while farmland expanded to 2.10 km2 (14.71 %). Open area constituted the smallest class in 2014, covering only 0.57 km2 (3.99 %).

By 2022, agroforestry retained its lead, encompassing 48.46 % (6.92 km2) of the total area. The built-up area experienced a significant increase, reaching 36.34 % (5.19 km2). Farmland decreased to 1.45 km2 (10.15 %), and open space remained the smallest class at 5.04 % (0.72 km2).

#### 3.2.2 SVM classification

**Fig. 6:**
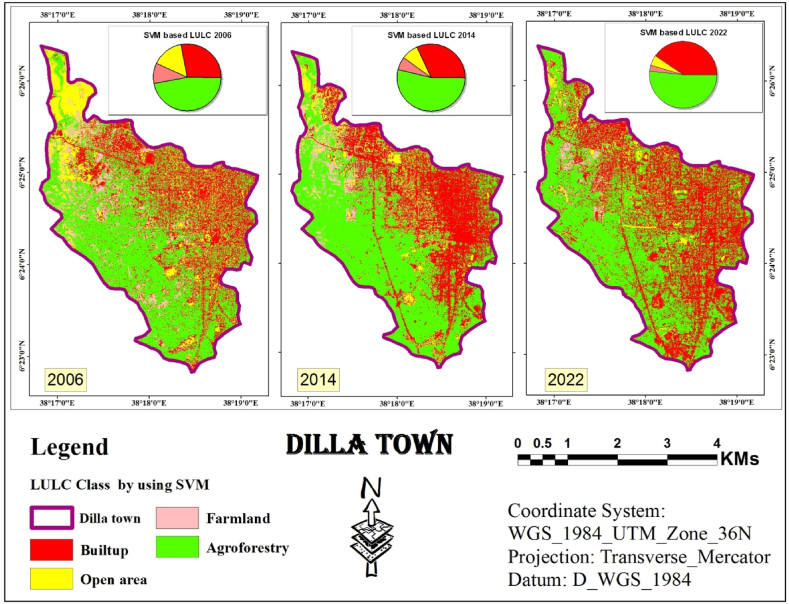
SVM classified image (2006, 2014 and 2022).

The study then leveraged the training data from the previous step to perform independent classifications for each year using SVM. SVM analyze data point features (e.g., groups of pixels) and assign them to the most likely class based on a trained model. To achieve the final classification, the segmented image from the first step was combined with the training data generated for each year using SVM (segment-based training models). Fig. 6 displays classified images obtained through SVM for the years 2006, 2014, and 2022. Finally, these classified images were assessed for accuracy using ArcMap.

The next step involved combining an object-based approach with SVM algorithm to classify LULC for the study area across three years: 2006, 2014, and 2022. Table 2 presents the calculated area results.

**Table 2:** Statistics of LULC classes using SVM Classifier.

LULC Class	2006		2014		2022	
	Area		Area		Area	
	(Km^2^)	%	(Km^2^)	%	(Km^2^)	%
Built-up	4.02	28.15	4.57	32	5.76	40.34
Open area	2.12	14.85	1.1	7.7	0.73	5.11
Farm land	1.42	9.94	0.92	6.44	0.41	2.87
Agroforestry	6.72	47.06	7.69	53.85	7.39	51.75
Total Area (Km^2^)	14.28	100	14.28	100	14.28	100

Based on the SVM classification outcomes derived from the 2006 assessment conducted in Dilla town, the predominant land cover class identified was agroforestry, encompassing an estimated 6.72 km2, accounting for 47.06 % of the total area. This class comprised various trees, shrubs, and scattered agricultural land with a low density of trees and shrubs. In contrast, the built-up area, the second most prevalent class, covered 4.02 Km^2^ which is 28.15 % of the area.

Open area ranked third, encompassing 2.12 Km^2^ or 14.85 % of the total area, likely resulting from the disappearance of farmland. Farmland was the least common class, totaling 1.42 Km^2^ or 9.94 % of the area.

In 2014 and 2022, agroforestry retained its dominance, covering 7.69 Km^2^ (53.85 %) and 7.39 Km^2^ (51.75 %) of the study area, respectively. Built-up areas remained the second largest class, occupying 4.57 Km^2^ (32 %) and 5.76 Km^2^ (40.34 %) in 2014 and 2022, respectively. Open areas declined to 1.10 Km^2^ (7.70 %) in 2014 and further to 0.73 Km^2^ (5.11 %) in 2022. Farmland followed a similar trend, decreasing to 0.92 Km^2^ (6.44 %) in 2014 and finally to 0.41 Km^2^ (2.87 %) in 2022.

#### 3.2.3 RF classification

**Fig. 7:**
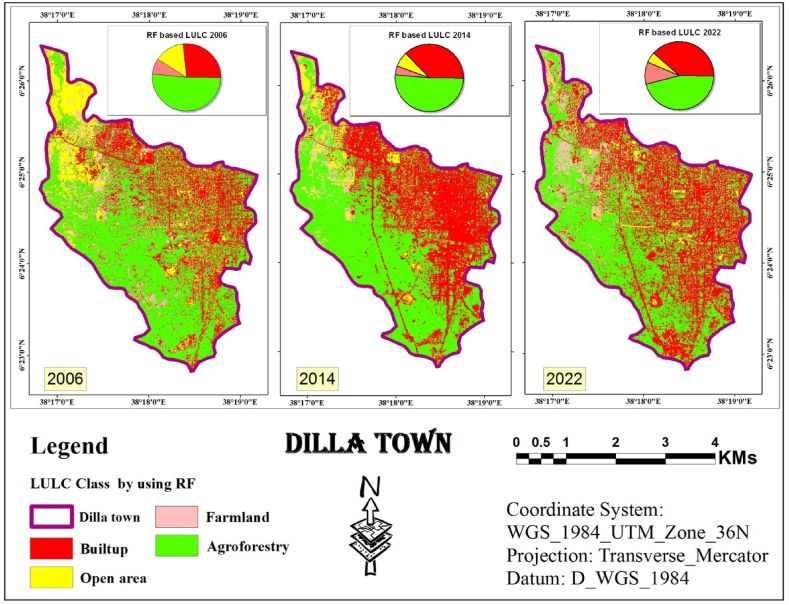
RF classified image (2006, 2014 and 2022).

RF is a popular choice in remote sensing due to its effectiveness, as demonstrated by various recent studies. It leverages two powerful techniques: bagging and random selection. These techniques are the core of the method. In this study, the random selection technique within ArcMap was employed to generate the LULC maps. Literature suggests using 200 decision trees for optimal results. Based on this recommendation, 200 decision trees were used to perform RF classification for the study area in the years 2006, 2014, and 2022 (presented in Fig. 7).

These decision trees are created by randomly selecting training samples and integrating them with object-based segmentation data. Importantly, feature variables are also randomly chosen to achieve the best possible splits within the trees. The final outputs were then analyzed in ArcMap for accuracy assessment and QGIS for change detection purposes.

The study used a third classification algorithm, RF, for LULC classification. To quantify the results and visualize the proportion of each class within the entire area, statistics were calculated for the final LULC map. The detailed statistical results are presented in Table 3.

**Table 3:** Statistics of LULC classes Using RF Classifier.

LULC Class	2006		2014		2022	
	Area		Area		Area	
	(Km^2^)	%	(Km^2^)	%	(Km^2^)	%
Built-up	3.83	26.82	5.32	37.25	5.49	38.45
Open area	2.02	14.15	1	7	0.73	5.11
Farmland	1.05	7.35	0.59	4.13	1.5	10.5
Agroforestry	7.38	51.68	7.37	51.61	6.57	46.01
Total Area (Km^2^)	14.28	100	14.28	100	14.28	100

According to RF-based classification the dominant land cover in the study area in 2006 (Fig. 7) was agroforestry, encompassing over half (51.68 %) of the area, or roughly 7.38 Km^2^. It included various trees, shrubs, and agricultural land with sparse vegetation. Built-up areas were the second most common, scattered throughout the study area and accounting for 26.82 % (3.83 Km^2^). Open areas ranked third at 14.15 % (2.02 Km^2^), followed by farmland (7.35 %, 1.05 Km^2^).

The ranking of land cover classes remained relatively stable in 2014. Agroforestry continued to dominate (51.61 %, 7.37 Km^2^), followed by built-up areas (37.25 %, 5.32 Km^2^). Open areas decreased to 7 % (1 Km^2^), while farmland became the smallest category (4.13 %, 0.59 Km^2^).

By 2022, agroforestry persisted as the leading land cover (46.01 %, 6.57 Km^2^). Built-up areas remained significant (38.45 %, 5.49 Km^2^). Farmland showed a slight increase (10.50 %, 1.50 Km^2^), while open areas became the smallest class (5.11 %, 0.73 Km^2^).

As information obtained from Dilla town land administration office, the study area underwent rapid urban sprawl between 2005 and 2014, followed by the implementation of a new urban plan in 2015. This plan involved the removal of low-standard settlements and their relocation to suburban areas. Additionally, settlements affected by new infrastructure projects, such as roads, green areas, telecom lines, and water pipelines, were partially cleared. By 2022, the central urban area had a lower density compared to 2014 due to these changes. Therefore, in the classified maps from 2014 generated by three algorithms (Fig. 5, Fig. 6, Fig. 7), there was a higher density of urban areas compared to both the 2006 and 2022 images.

### 3.3 Accuracy assessment

Although remotely sensed imagery allows for the creation of LULC maps, these maps can contain inaccuracies. To ensure the maps are reliable for further analysis and to gauge the effectiveness of the machine learning algorithms used for classification, an accuracy assessment is crucial.

Various quantitative measures are employed to assess the performance of the trained machine learning models (ANN, SVM, and RF). Overall accuracy, kappa coefficient, Producer's accuracy and User's accuracy are the most common methods.

For a complete evaluation, the classified maps need to be compared to a reference dataset, also known as ground truth. This comparison is typically done using an error matrix, which highlights areas of agreement and disagreement between the map and the reference data.

Fig. 5, Fig. 6, Fig. 7 shows the identification and mapping of four main urban LULC types for 2006, 2014, and 2022 periods.

#### 3.3.1 ANN based classification accuracy assessment result

As indicated in Table 4, ANN was used to classify land cover data for the years 2006, 2014, and 2022. The overall accuracy of the classification was 86 %, 85 %, and 96 %, respectively. Additionally, the Kappa statistic, which measures agreement beyond random chance, also showed good results, with values of 0.74, 0.75, and 0.93 for the respective years. Furthermore, for the built-up class, user's accuracy was 0.93, 0.91, and 0.97 in 2006, 2014, and 2022, respectively, while producer's accuracy was 0.86, 0.81, and 0.96 for the corresponding years.

**Table 4:** Overall accuracy, kappa statistic, User's accuracy (built-up), Producer's accuracy (built-up) results of ANN, SVM and RF classifiers for the images of 2006, 2014 and 2022.

Image (Year)	Method	Overall Accuracy	Kappa Statistic	User's Accuracy (Built-up)	Producer's Accuracy (Built-up)
2006	ANN	86 %	0.74	0.93	0.86
2014	ANN	85 %	0.75	0.91	0.81
2022	ANN	96 %	0.93	0.97	0.96
2006	SVM	95 %	0.97	0.99	0.97
2014	SVM	96 %	0.97	0.98	0.96
2022	SVM	96 %	0.98	0.98	0.97
2006	RF	96 %	0.98	0.99	0.98
2014	RF	97 %	0.98	0.98	0.97
2022	RF	97 %	0.98	0.99	0.97

#### 3.3.2 SVM based classification accuracy assessment result

Similar to the ANN approach, SVM was used to classify land cover data for the years 2006, 2014, and 2022. As shown in Table 4, the SVM achieved very high overall accuracy, reaching 95 %, 96 %, and 96 % for the respective years. The Kappa statistics further supported this, with values of 0.97, 0.97, and 0.98 for the corresponding years, indicating strong agreement beyond random chance. Additionally, for the built-up class, user's accuracy was exceptionally high at 0.99, 0.98, and 0.98 in 2006, 2014, and 2022, respectively. Producer's accuracy also remained impressive, reaching 0.97, 0.96, and 0.97 for the corresponding years.

#### 3.3.3 RF based classification accuracy assessment result

RF based classification algorithm delivered impressive results. As depicted in Table 4, the overall accuracy for the years 2006, 2014, and 2022 were all exceptionally high, at 96 %, 97 %, and 97 % respectively. This strong performance is further corroborated by the Kappa statistics, which were consistently high at 0.98 for all three years. Furthermore, similar to SVM, the user's accuracy for the built-up class achieved remarkable results, reaching 0.99, 1.00, and 0.99 in 2006, 2014, and 2022, respectively. Producer's accuracy was also very good, with values of 0.98, 0.97, and 0.97 for the corresponding years.

### 3.4 Comparison of accuracy assessment of ANN, SVM and RF

**Fig. 8:**
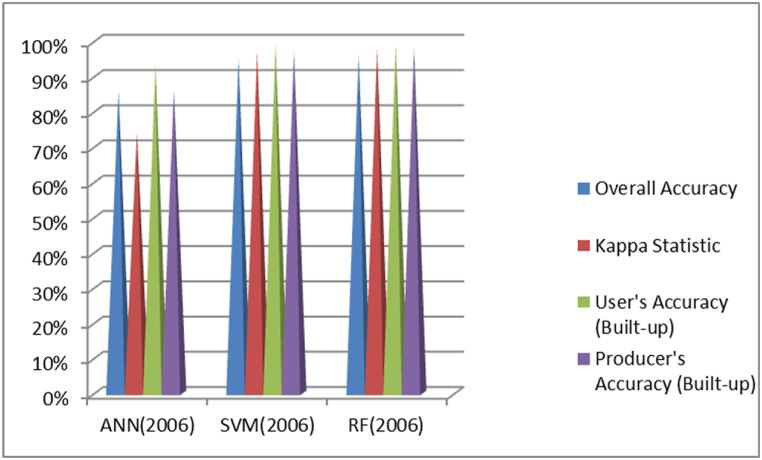
Comparison of ANN, SVM and RF based classification overall accuracy and kappa coefficient result of 2006.

As indicated in Fig. 8, in the classification of the 2006 image, RF achieved the highest Overall Accuracy at 96 %, followed by SVM at 95 % and ANN at 86 %. When considering Kappa Statistic, which measures agreement beyond overall accuracy, RF scored the highest at 0.98, followed by SVM at 0.97 and ANN at 0.74. For User's Accuracy (Built-up), both RF and SVM had the highest accuracy at 0.99, with ANN slightly lower at 0.93. Producer's Accuracy (Built-up) was also highest for RF and SVM at about 0.98 and 0.97 respectively, while ANN lagged at 0.86. Overall, SVM performed the best in this classification. Based on these metrics, the RF classifier appears to perform the best overall. Particularly it has the highest Overall Accuracy, Kappa Statistic and Producer's Accuracy.

**Fig. 9:**
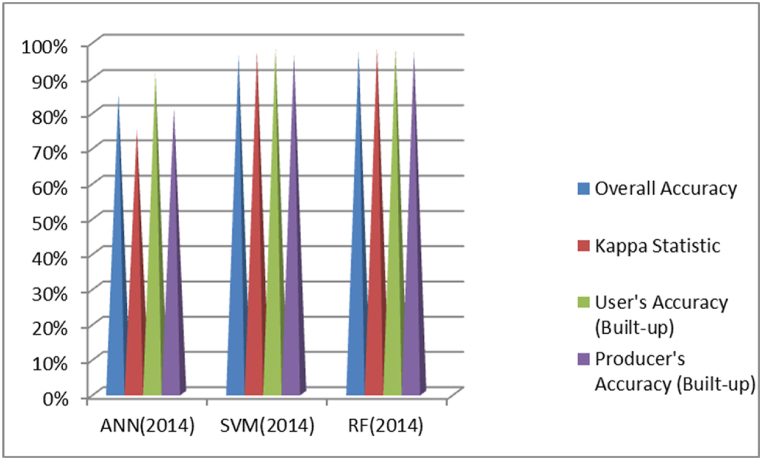
Comparison of ANN, SVM and RF based classification overall accuracy and kappa coefficient result of 2014.

As shown in Fig. 9, for the 2014 images, RF achieved the highest Overall Accuracy at 97 %, followed closely by SVM at 96 % and ANN at 85 %. In terms of Kappa Statistic, both SVM and RF scored the highest at 0.98, while ANN had a lower score of 0.75. User's Accuracy (Built-up) was tied between RF and SVM at 0.98, with ANN at 0.91. Producer's Accuracy (Built-up) was again highest for SVM and RF at around 0.97, while ANN was at 0.81. Overall, RF showed the best performance in this classification. Based on these metrics, the RF classifier appears to perform the best overall. It has the highest Overall Accuracy and is tied with SVM for the highest Kappa Statistic. While SVM is similar in most metrics, RF has a slight edge in overall accuracy.

**Fig. 10:**
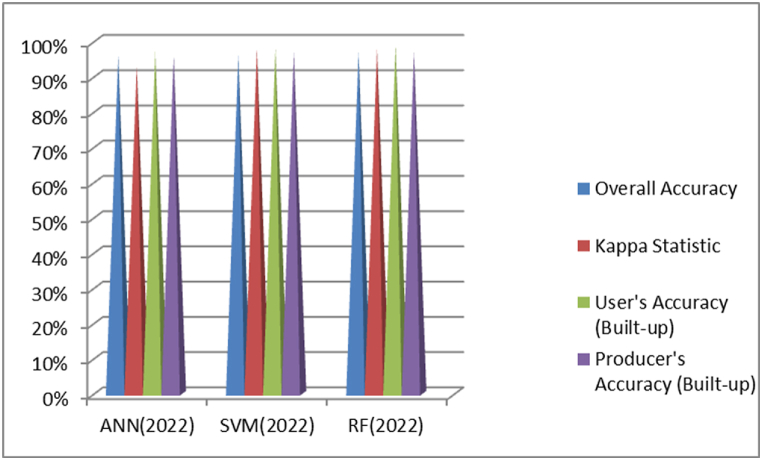
Comparison of ANN, SVM and RF based classification overall accuracy and kappa coefficient result of 2022.

As depicted in Fig. 10, for the 2022 images, RF once again achieved the highest Overall Accuracy at 97 %, followed by SVM and ANN, both at 96 %. In terms of Kappa Statistic, both SVM and RF scored the highest at 0.98, while ANN had a score of 0.93. User's Accuracy (Built-up) was highest for RF at 0.99, followed by SVM at 0.98 and ANN at 0.97. Producer's Accuracy (Built-up) was similar for all three classifiers at around 0.97. Overall, RF outperformed the other classifiers in this classification as well.

Generally, RF demonstrated superior performance across all three image classifications based on Overall Accuracy, Kappa Statistic, User's Accuracy, and Producer's Accuracy metrics.

### 3.5 Comparison of ANN, SVM and RF based on computation time, algorithm stability and visual quality

While accuracy is crucial, other factors influence the choice of a machine learning algorithm for LULC classification. These include computation time, algorithm stability, and the visual quality of the output. This study examines all these aspects for a comprehensive comparison.

#### 3.5.1 Computation time

**Fig. 11:**
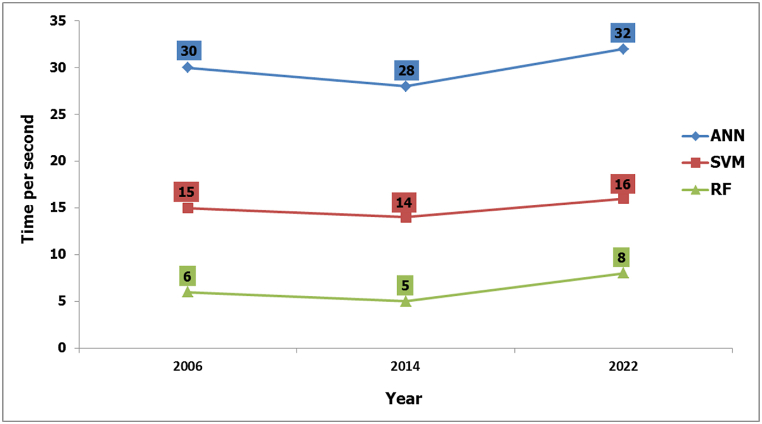
Total processing time of ANN, SVM and RF for 2006, 2014 and 2022 image.

As accuracy, understanding how fast an algorithm processes data is important. Here, the study compared the computation time of three popular choices: ANN, SVM, and RF (Fig. 11).

In general, training all three algorithms took only a few seconds regardless of the data size. However, when looking closer at processing speed, the analysis revealed that ANN took the longest time, followed by SVM. In contrast, RF classifications were significantly faster. This is because, unlike ANN which transforms data into a higher-dimensional space, RF builds multiple, simpler decision trees with boundaries parallel to the existing feature axes [38].

#### 3.5.2 Algorithm stability

The study investigated how the size of training data affects the performance of different classifiers for land cover mapping. While all classifiers showed improved accuracy with more training data, ANN, SVM, and RF displayed more stable performance across various training data sizes.

This stability likely stems from the inherent strengths of each method. ANN benefit from weighted connections and non-linear activation functions that adjust values within the network. SVM use a strategy to minimize errors on unseen data without needing assumptions about data distribution. RF introduces diversity by re-sampling the training data for each tree, reducing the impact of outliers and leading to less classification error.

#### 3.5.3 Visual assessment

**Fig. 12:**
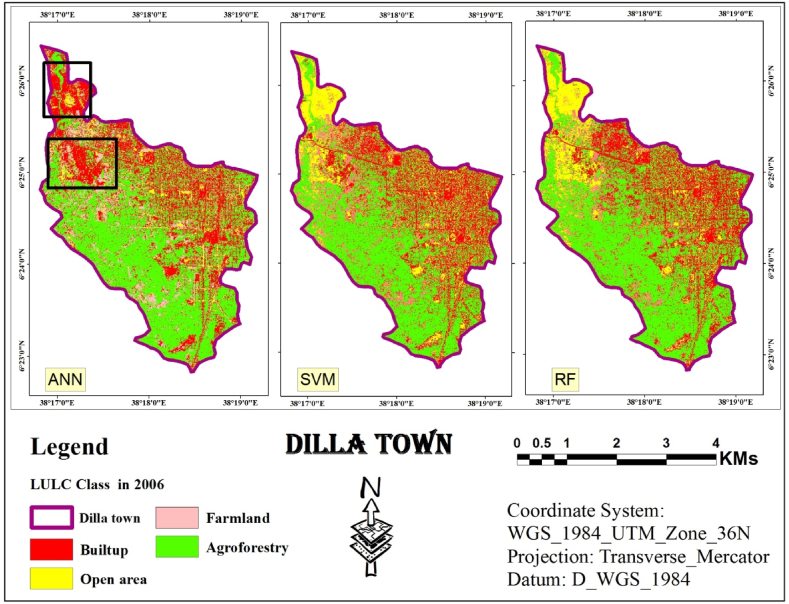
LULC map of 2006 based on ANN, SVM and RF.

The visual assessment confirmed these findings. Land cover maps generated by each classifier (Fig. 5, Fig. 6, Fig. 7) showed minimal distortion compared to actual observations (Fig. 12). This aligns with the statistical accuracy results, suggesting all three classifiers performed well in capturing land cover information. ”

Using the study area image of 2006, a maps generated using an ANN classifier, as shown in Fig. 12, successfully captured the general layout of the study area. However, these maps contained numerous errors, particularly in the northern portion where open areas were frequently misclassified as urban areas. Ground observations confirmed these misclassifications.

Conversely, classifiers based on SVM and RF produced maps with significantly less distortion. Additionally, these SVM and RF-derived maps exhibited greater homogeneity within each land use/cover class.

**Fig. 13:**
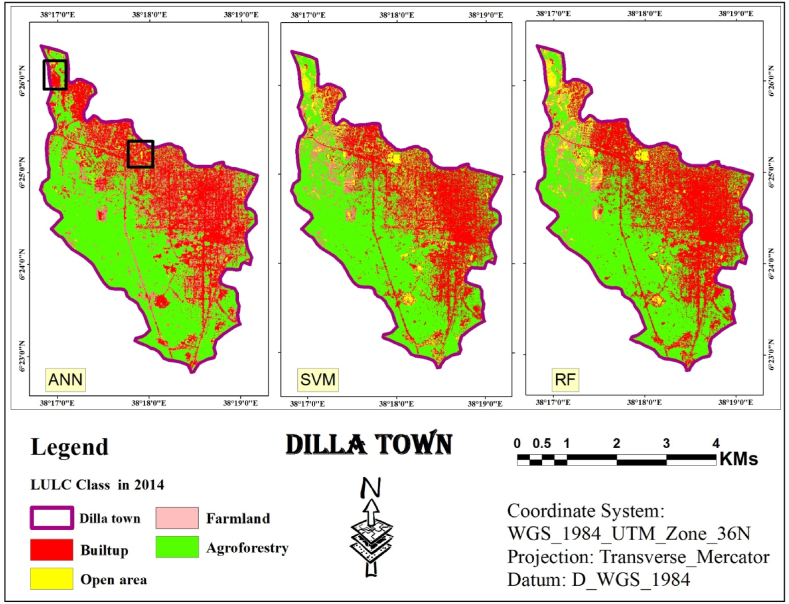
LULC map of 2014 based on ANN, SVM and RF.

A comparison of the maps created using an image of 2014 using the three classification algorithms reveals some interesting findings (Fig. 13). The map generated by the ANN classifier shows errors in the northern and northwestern regions. In particular, there is confusion between “Farmland” and “Built-up” areas, as well as between “Built-up” and “Open area."

While the RF and SVM classifiers produce maps with less distortion, there are still significant differences between the RF and ANN algorithms. Based on observations of the area, the RF classifier appears to have the most accurate visual representation overall.

**Fig. 14:**
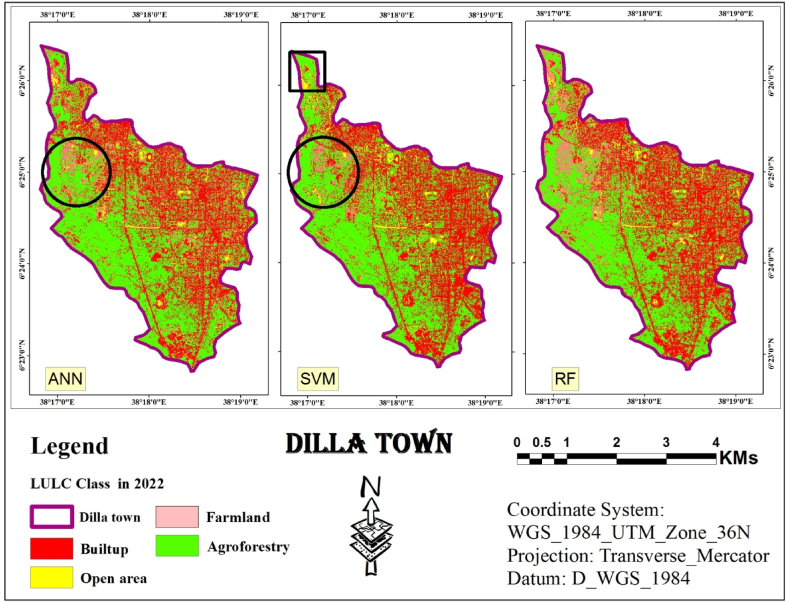
LULC map of 2022 based on ANN, SVM and RF.

Using the image of 2022, the classifiers algorism successfully assigned most areas to their corresponding classes, as illustrated in Fig. 14. However, some errors persisted, particularly between “Agroforestry” and “Farmland” due to their similar features. The SVM classifier also showed errors in the north-western region, misclassifying “Farmland” as “Built up” as confirmed by ground observations.

While both SVM and RF classifiers reduced errors compared to the ANN classifier, there was little overall difference in performance between these two algorithms. However, RF produced maps with visually clearer class distinctions.

### 3.6 Change detection

**Fig. 15:**
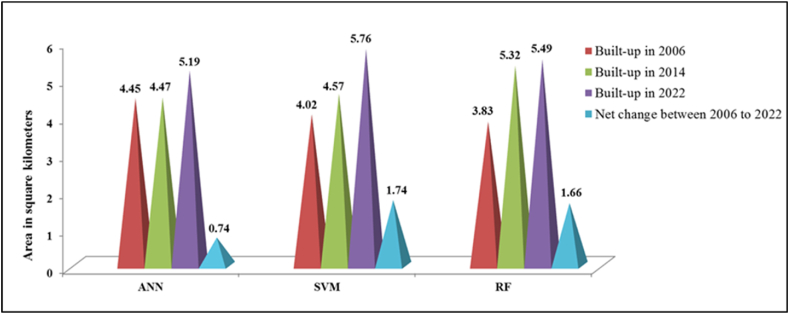
Comparison of Built-up area change detection by different algorithms (ANN, SVM and RF) for the years 2006, 2014, and 2022.

Fig. 15 bellow shows the built-up area change detection using different algorithms (ANN, SVM and RF) for the years 2006, 2014, and 2022. In 2006, the ANN algorithm detected a built-up area of 4.45 Km^2^, SVM detected 4.02 Km^2^, and RF detected 3.83 Km^2^. By 2014, the built-up area increased to 4.47 Km^2^ for ANN, 4.57 Km^2^ for SVM, and 5.32 Km^2^ for RF. In 2022, the built-up area further increased to 5.19 Km^2^ for ANN, 5.76 Km^2^ for SVM, and 5.49 Km^2^ for RF. The net change in built-up area between 2006 and 2022 was increased by 0.74 Km^2^ for ANN, 1.74 Km^2^ for SVM, and 1.66 Km^2^ for RF. These results showcase the variations in detecting changes in built-up areas over the specified years using different algorithms.

The comparison result reveals interesting insights. The ANN algorithm consistently detects a relatively small amount of net change, with a modest increase of 0.74 Km^2^ from 2006 to 2022. This suggests that the ANN algorithm may be more conservative in detecting changes in built-up areas compared to the other algorithms. On the other hand, the SVM algorithm tends to detect a little exaggerated net change, showing larger variations in built-up areas with a net change of increase by 1.74 Km^2^ over the same period. This indicates that SVM may be more sensitive to fluctuations in built-up areas, potentially capturing both minor and significant changes in the landscape. In contrast, the RF algorithm demonstrates a remarkable ability to detect relatively precise net changes in built-up areas, with a net change of increase by 1.66 Km^2^ from 2006 to 2022. The high precision in detecting areal coverage between 2006 and 2022, along with the net change detection, aligns with the data obtained (increased by 1.65 Km^2^ between 2006 and 2022) from the Dilla town land administration office.

This study acknowledges limitations due to its scope. While it provides valuable insights using machine learning with object-based classifiers, future work can delve deeper. Firstly, exploring pixel-based integration alongside the current method would allow performance comparisons between the two approaches. Secondly, the study's focus on a flat urban area limits generalizability. Testing in large, rough terrains would broaden the applicability of the findings. Thirdly, the current investigation into overall land cover types could be expanded to assess performance on specific types in future studies.

## 4 Conclusion

This study compared the effectiveness of three machine learning algorithms –ANN, SVM, and RF – for mapping and detecting LULC changes in Dilla town, Ethiopia, between 2006 and 2022. The imagery data was utilized from three specific years: 2006, 2014, and 2022. The primary objective was to identify the most accurate and reliable technique for LULC classification of the study area. Four main LULC categories were identified for analysis: built-up areas, open spaces, farmland, and agroforestry.

The evaluation of the algorithms considered several factors, including overall accuracy, kappa coefficient, producer's accuracy, user's accuracy, computational time, algorithm stability, and visual quality. The results demonstrated that the RF algorithm outperformed the others in all evaluation criteria. The RF classifier achieved superior performance with an average overall accuracy of 0.97, kappa coefficient of 0.98, producer's accuracy of 0.99, and user's accuracy of 0.97. This is demonstrably better than both SVM (0.96, 0.97, 0.98, and 0.97) and ANN (0.89, 0.81, 0.94, and 0.88). These findings align with previous research highlighting the accuracy of RF for LULC classification [[39], [40], [41]].

Furthermore, RF training was significantly faster than both SVM and ANN. On average, RF training required only 6.33 s, while SVM and ANN took 15 and 30 s respectively, for processing similar data sizes. This efficiency aligns with prior studies showcasing RF's advantages for LULC mapping [8,42].

In terms of stability, all three algorithms exhibited consistent performance across varying training data sizes. This aligns with existing research on the stability of ANN, SVM, and RF [[43], [44], [45]]. Additionally, a visual inspection revealed minor variations in the precision of each classifier, with RF displaying the highest visual quality. This is consistent with previous studies suggesting that RF produces the most interpretable results for visual analysis [[46], [47], [48]].

The built-up change detection result shows the net change in built-up area between 2006 and 2022 was increase by 0.74 Km^2^ for ANN, 1.74 Km^2^ for SVM, and 1.66 Km^2^ for RF. The comparison reveals that the ANN algorithm shows a relatively small net change, indicating a conservative approach. In contrast, the SVM algorithm demonstrates a tendency to detect exaggerated net changes, reflecting sensitivity to fluctuations. The RF algorithm stands out for its precise net change detection, showcasing high precision in detecting areal coverage over the years, consistent with the data obtained from Dilla town land administration office.

A crucial area recommended for further investigation is the exploration of factors that impact the performance of classification algorithms, as well as the development of methodologies to enhance the overall efficacy of machine learning algorithms.

## CRediT authorship contribution statement

**Melion Kasahun:** Writing – original draft, Software, Resources, Methodology, Formal analysis, Data curation, Conceptualization. **Abiyot Legesse:** Writing – review & editing, Supervision.

## Ethics approval

Not applicable.

## Availability of data and material

The datasets used and/or analyzed during the current study are available in the article/from the corresponding author on request.

## Funding

This research received no funding from any source.

## Declaration of competing interest

The authors declare that they have no known competing financial interests or personal relationships that could have appeared to influence the work reported in this paper.

## References

[1] Guan D., Li H., Inohae T., Su W., Nagaie T., Hokao K. (2011). Modeling urban land use change by the integration of cellular automaton and Markov model. Ecol. Model..

[2] Satterthwaite D., McGranahan G., Tacoli C. (Sep. 2010). Urbanization and its implications for food and farming. Philos. Trans. R. Soc. B Biol. Sci..

[3] Wogderes A. (2014). http://etd.aau.edu.et/handle/123456789/4797.

[4] Zamani A., Sharifi A., Felegari S., Tariq A., Zhao N. (Jan. 2022). Agro climatic zoning of saffron culture in miyaneh city by using WLC method and remote sensing data. Agriculture.

[5] Wassie S.B. (Nov. 2020). Natural resource degradation tendencies in Ethiopia: a review. Environ. Syst. Res..

[6] Buriánek D. Explanatory notes to the thematic geoscientific maps of Ethiopia at a scale of 1 : 50,000 Map Sheet 0638-C2 Dila. http://www.geology.cz/etiopie-2018/outputs/dila/explanatory-notes-0638-c2-dila.pdf.

[7] Sharifi Alireza (2020). Remotely sensed vegetation indices for crop nutrition mapping. J. Sci. Food Agric..

[8] Raczko E., Zagajewski B. (Jan. 2017). Comparison of support vector machine, random forest and neural network classifiers for tree species classification on airborne hyperspectral APEX images. Eur. J. Remote Sens..

[9] Sanlı T., Sıcakyüz Ç., Yüregir O.H. (Sep. 2020). Comparison of the accuracy of classification algorithms on three data-sets in data mining: example of 20 classes. Int. J. Eng. Sci. Technol..

[10] J. Nelson and 2020 6 Min Read, “Why should I do pre-processing and augmentation on my computer vision datasets?,” Roboflow Blog. Accessed: August. 16, 2022. [Online]. Available: https://blog.roboflow.com/why-preprocess-augment/.

[11] Sharifi A., Amini J., Sri Sumantyo J., Tateishi R. (Jun. 2015). Speckle reduction of PolSAR images in forest regions using fast ICA algorithm. J. Indian Soc. Remote Sens..

[12] Canada N.R. Image enhancement. https://www.nrcan.gc.ca/maps-tools-and-publications/satellite-imagery-and-air-photos/tutorial-fundamentals-remote-sensing/image-interpretation-analysis/image-enhancement/9389.

[13] Pourasad Y., Cavallaro F. (Jun. 2021). A novel image processing approach to enhancement and compression of X-ray images. Int. J. Environ. Res. Publ. Health.

[14] Sahidan S.I., Mashor M.Y., Wahab A.S.W., Salleh Z., Ja’afar H., Abu Osman N.A., Ibrahim F., Wan Abas W.A.B., Abdul Rahman H.S., Ting H.-N. (2008). 4th Kuala Lumpur International Conference on Biomedical Engineering 2008.

[15] Das T. (2009). https://www.semanticscholar.org/paper/Land-use-%2F-land-cover-change-detection%3A-an-object-Das/15ecbae2e4a2ebcaaa3bd2eac93ad30b098145b4.

[16] Baysal G. (Jan. 2013). Urban land use and land cover change analysis and modeling a case study area Malatya, Turkey. https://run.unl.pt/handle/10362/9187?locale=en.

[17] Shapiro L.G., Stockman G.C. (2001).

[18] Hajdowska K., Student S., Borys D. (Jan. 2022). Graph based method for cell segmentation and detection in live-cell fluorescence microscope imaging. Biomed. Signal Process Control.

[19] Reinhardt M. (Jan. 2022). Benchmarking conventional and machine learning segmentation techniques for digital rock physics analysis of fractured rocks. Environ. Earth Sci..

[20] Yogamangalam R., Karthikeyan B. (Jan. 2013). Segmentation techniques comparison in image processing. Int. J. Eng. Technol..

[21] Xiao W., Zaforemska A., Smigaj M., Wang Y., Gaulton R. (Jan. 2019). Mean shift segmentation assessment for individual forest tree delineation from airborne lidar data. Rem. Sens..

[22] Lu C. (2019). https://www.semanticscholar.org/paper/Object-based-Classification-of-High-Spatial-Remote-Lu/9458e51b943a2e74d65a3846a7be43415260fc08.

[23] Zhou H., Wang X., Schaefer G., Kwaśnicka H., Jain L.C. (2011). Innovations in Intelligent Image Analysis.

[24] Esri Segmenting an image. https://pro.arcgis.com/en/pro-app/2.7/help/analysis/image-analyst/segmentation.htm.

[25] Sharifi A., Hosseingholizadeh M. (2019). Application of sentinel-1 data to estimate height and biomass of rice crop in astaneh-ye ashrafiyeh, Iran. J. Indian Soc. Remote Sens..

[26] Farmonov N. (Jan. 2023). Crop type classification by DESIS hyperspectral imagery and machine learning algorithms. IEEE J. Sel. Top. Appl. Earth Obs. Rem. Sens..

[27] Du P., Samat A., Waske B., Liu S., Li Z. (2015). Random Forest and Rotation Forest for fully polarized SAR image classification using polarimetric and spatial features. ISPRS J. Photogrammetry Remote Sens..

[28] Bryden J. Biologically inspired computing: the neural network. https://www.academia.edu/35380032/Biologically_Inspired_Computing_The_Neural_Network.

[29] Xiong Y., Zhang Z., Chen F. (Nov. 2010). Presented at the ICCASM 2010 - 2010 International Conference on Computer Application and System Modeling, Proceedings.

[30] Uba N.K. (May 2019). Land use and land cover classification using deep learning techniques. https://ui.adsabs.harvard.edu/abs/2019arXiv190500510U.

[31] Mira J., Sandoval F. (1995).

[32] Grenier M., Labrecque S., Benoit M., Allard M. (Jan. 2008). Proc. GEOBIA 2008 - Pixels Objects Intell. Geogr. Object Based Image Anal. 21st Century.

[33] Lu D., Weng Q. (Mar. 2007). A survey of image classification methods and techniques for improving classification performance. Int. J. Rem. Sens..

[34] DeFries R.S., Chan J.C.W. (2000). Multiple criteria for evaluating machine learning algorithms for land cover classification from satellite data. Remote Sens. Environ..

[35] Esmaeili M., Abbasi-Moghadam D., Sharifi A., Tariq A., Li Q. (2023). Hyperspectral image band selection based on CNN embedded ga (CNNeGA). IEEE J. Sel. Top. Appl. Earth Obs. Rem. Sens..

[36] Campbell J.B., Wynne R.H. (2011). Introduction to Remote Sensing.

[37] Paiboonvorachat Chamaporn Using remote sensing and GIS techniques to assess land use/land cover changes in the Nan watershed, Thailand. https://www.proquest.com/openview/3b61f687166497d86800efcef4531efb/1?pq-origsite=gscholar&cbl=18750.

[38] Jin J. (2012).

[39] Chen R.-C., Dewi C., Huang S.-W., Caraka R.E. (Jul. 2020). Selecting critical features for data classification based on machine learning methods. J. Big Data.

[40] Fawagreh K., Gaber M.M., Elyan E. (Dec. 2014). Random forests: from early developments to recent advancements. Syst. Sci. Control Eng..

[41] Song J. (Mar. 2021). “<p>The random forest model has the best accuracy among the four pressure ulcer prediction models using machine learning algorithms</p>,”. Risk Manag. Healthc. Pol..

[42] Petropoulos G.P., Kontoes C.C., Keramitsoglou I. (Aug. 2012). Land cover mapping with emphasis to burnt area delineation using co-orbital ALI and Landsat TM imagery. Int. J. Appl. Earth Obs. Geoinformation.

[43] Boateng E.Y., Otoo J., Abaye D.A. (Sep. 2020). Basic tenets of classification algorithms K-Nearest-Neighbor, support vector machine, random forest and neural network: a review. J. Data Anal. Inf. Process..

[44] Rodriguez-Galiano V., Chica Rivas M. (2012). Evaluation of different machine learning methods for land cover mapping of a Mediterranean area using multi-seasonal Landsat images and Digital Terrain Models. Int. J. Digit. Earth.

[45] Zahra M., Essai Ali M.H., Refaee A. (Dec. 2013). Robust neural network Classifier| ISSN: 2321-9939. Int. J. Eng. Dev. Res. IJEDR.

[46] Alshari E., Gawali B. (2021). Development of classification system for LULC using remote sensing and GIS. Glob. Transit. Proc..

[47] Wu C. (Sep. 2020). An automated machine-learning approach for road pothole detection using smartphone sensor data. Sensors.

[48] Yang Y., Yang D., Wang X., Zhang Z., Nawaz Z. (Jan. 2021). Testing accuracy of land cover classification algorithms in the qilian mountains based on GEE cloud platform. Rem. Sens..

